# Oncological Outcomes After Hippocampus-Sparing Whole-Brain Radiotherapy in Cancer Patients With Newly Diagnosed Brain Oligometastases: A Single-Arm Prospective Observational Cohort Study in Taiwan

**DOI:** 10.3389/fonc.2021.784635

**Published:** 2022-01-12

**Authors:** Shinn-Yn Lin, Din-Li Tsan, Chi-Cheng Chuang, Chi-Cheng Yang, Ping-Ching Pai, Chih-Liang Wang, Yi-Ming Wu, Cheng-Chi Lee, Chia-Hsin Lin, Kuo-Chen Wei, Wen-Chi Chou

**Affiliations:** ^1^ Department of Radiation Oncology, Chang Gung Memorial Hospital and Chang Gung University, Taoyuan, Taiwan; ^2^ Department of Medical Imaging and Radiological Sciences, College of Medicine, Chang Gung University, Taoyuan, Taiwan; ^3^ Department of Neurosurgery, Chang Gung Memorial Hospital and Chang Gung University, Taoyuan, Taiwan; ^4^ College of Medicine, Chang Gung University, Taoyuan, Taiwan; ^5^ Department of Psychology, National Chengchi University, Taipei, Taiwan; ^6^ Department of Thoracic Medicine, Chang Gung Memorial Hospital, Taoyuan, Taiwan; ^7^ Department of Medical Imaging and Intervention, Chang Gung Memorial Hospital and Chang Gung University, Taoyuan, Taiwan; ^8^ School of Medicine, National Yang-Ming University, Taipei, Taiwan; ^9^ Deparment of Hematology-Oncology, Chang Gung Memorial Hospital and Chang Gung University, Taoyuan, Taiwan

**Keywords:** brain oligometastases, oncological outcomes, competing risks, hippocampus-sparing whole-brain radiotherapy, neurologic death

## Abstract

**Background:**

Promisingly, the technique of hippocampus sparing during WBRT (HS-WBRT) might preserve NCFs. In this research, we examined oncological outcomes, with emphasis on neurologic/non-neurologic causes of death, CNS progression, and leptomeningeal disease (LMD) recurrence in cancer patients who underwent HS-WBRT.

**Methods:**

One hundred and fourteen cancer patients with newly diagnosed brain oligometastases underwent HS-WBRT were consecutively enrolled. The cumulative incidence of cancer-specific deaths (neurologic or non-neurologic), LMD recurrence, and the composite endpoint of CNS progression (CNS-CE) as the first event were computed with a competing-risks approach to characterize the oncological outcomes after HS-WBRT.

**Results:**

Patients with intact brain metastases had a significantly increased likelihood of dying from non-neurologic causes of death associated with early manifestation of progressive systemic disease (hazard ratio for non-neurologic death, 1.78; 95% CI, 1.08–2.95; *p* = 0.025; competing-risks Fine–Gray regression), which reciprocally rendered them unlikely to encounter LMD recurrence or any pattern of CNS progression (HR for CNS-CE as the first event, 0.13; 95% CI, 0.02–0.97; *p* = 0.047; competing-risks Fine–Gray regression). By contrast, patients with resection cavities post-craniotomy had reciprocally increased likelihood of CNS progression which might be associated with neurologic death eventually.

**Conclusions:**

Patterns of oncological endpoints including neurologic/non-neurologic death and cumulative incidence of CNS progression manifesting as LMD recurrence are clearly clarified and contrasted between patients with intact BMs and those with resection cavities, indicating they are clinically distinct subgroups.

**Trial Registration:**

ClinicalTrials.gov, Identifier: NCT02504788, NCT03223675.

## Introduction

Approximately 20%–40% of patients with brain cancer have brain metastasis (BM), resulting in poor prognosis (1). Owing to advances in diagnosis and treatment, many patients with BM, a common adult intracranial neoplasm, are surviving longer. Traditionally, whole-brain radiation therapy (WBRT), with or without surgical resection, might be the treatment of choice for managing BMs.

According to the latest treatment guidelines of the National Comprehensive Cancer Network (2), surgical resection improves oncological outcomes in cancer patients with newly diagnosed BMs. Alternatively, stereotactic radiotherapy (SRS) can be used in patients with oligometastatic brain disease and surgery-related morbidities. Most patients with multiple and disseminated BM lesions can be effectively managed initially with conventional WBRT without special requirements for conformal radiotherapy in real-world practice.

The hippocampus plays a vital role in maintaining memory functions (3). Brain irradiation, particularly WBRT, is detrimental to hippocampal neurogenesis (4), and impaired stem-cell neurogenesis is strongly associated with cognitive dysfunction (5). Moreover, isodose distribution specific to the hippocampus is negatively correlated with neuropsychological performance in patients receiving cranial irradiation for treating primary brain tumors (6). Therefore, hippocampus sparing during WBRT (HS-WBRT) for managing brain oligometastases has preserved neurocognitive functions (NCFs) (7). Besides, the use of volumetric modulated arc therapy or helical tomotherapy provides conformal sparing of the centrally located hippocampus while delivering uniform dose to the remaining brain parenchyma (8).

To better characterize survival outcomes in cancer patients with oligometastatic brain disease after receiving HS-WBRT, clarifying the cancer-specific causes of death and attributing them to neurologic or non-neurologic cause will assist neuro-oncologists in prioritizing and escalating the intensity of intracranial and extracranial therapies (9, 10). Additionally, various neuro-oncological outcomes, including the cumulative incidence of leptomeningeal disease (LMD) after brain irradiation, were explored thoroughly taking into account the competing risks of death or a mutually exclusive cancer-specific cause of death (neurologic/non-neurologic) (11, 12). Herein, this long-term follow-up report aims mostly to explore the meaningful influences of patient-, disease-, and treatment-related variables on the risk of neurologic death, or non-neurologic death, and oncological outcomes with particular emphasis on CNS progression.

## Methods

### Patient Selection

In this prospective study, 114 brain irradiation-naive cancer patients were consecutively enrolled between March 2013 and December 2020. The study protocol was approved by the Institutional Review Board at our institute (IRB 101-4151B and 103-1090C), and all participants provided written informed consent. The recruited patients were adult cancer patients with radiotherapy-naive brain oligometastases and a fair/good performance status. All recruited patients had a limited BM disease burden, termed oligometastatic brain disease, as previously described by us (8).

### Pretreatment Evaluations and Clinical Follow-Up

To ensure no detectable BM within a 5-mm margin around either hippocampus, all participants received gadolinium-enhanced MRI within 1 month before undergoing HS-WBRT, regardless of whether they had received preceding neurosurgical resection. Furthermore, all enrolled participants underwent baseline neurocognitive assessment within 2 weeks before the start of HS-WBRT.

After the standardized course of HS-WBRT, adhering to our study protocol (8), was administered, brain MRI examination was carried out at 4 and 12 months after HS-WBRT. All recruited patients were regularly followed up and surveyed for detecting the occurrence of CNS progression/failure suspected radiographically and clinically throughout the study period. Three patterns of CNS progression/failure were classified: intracranial local failure (LF), distant brain parenchymal failure (DBF), and development of LMD. All the enrolled patients were followed up until death or June 1, 2021.

### Statistical Considerations and Analyses

Various oncological outcomes in addition to overall survival (OS) were evaluated, with special emphasis on the cumulative incidence of several neuro-oncological endpoints, including neurologic/non-neurologic death (9, 10), LMD recurrence, and CNS progression as the first event (13). The events in relation to CNS progression were measured as a composite endpoint (CNS-CE), comprising three patterns of CNS progression: local recurrence, distant brain recurrence, and LMD. These three patterns of CNS progression/failure can manifest individually, synchronously, or metachronously. CNS progression is defined as radiographic/clinical evidence of progressively enlarging brain metastatic lesion(s) or any clinical situation suggesting the occurrence of LMD.

With death as a competing risk, the cumulative incidence probability of CNS progression as the first event (13) was computed with a competing-risks approach (12), with statistical values adjusted for the competing risks of death or pre-existing non-CNS progression. The effects of multiple covariates and potentially meaningful confounding factors were investigated with the Fine–Gray proportional hazards model (12). The Statistical Package for Social Sciences, version 20.0 (SPSS, Chicago, IL, USA) was used for statistical analyses.

## Results

### Patient Characteristics

A total of 114 patients were analyzed in this prospective study, with a median follow-up of 19.6 months (range, 0.43–95.0) for all consecutively recruited patients and 36.2 months (range, 10.5–95.0) for the surviving patients. These 114 patients were further categorized into two clinical subcohorts depending on whether upfront craniotomy plus tumor resection was performed, with surgical cavities post-craniotomy (*n* = 85) and without (*n* = 29).

The demographics and clinical characteristics of all 114 patients are listed in **Table 1**. As shown in **Table 1**, 54 males (47.4%) and 60 females (52.6%) were enrolled, with a median age of 57.3 years at registration. Most participants (81.6%) had a fair/satisfactory performance status (ECOG performance status 0–1). Fewer patients had a favorable performance status (KPS 90 or better) in the subcohort undergoing recent craniotomy plus tumor removal. The majority of the participants had primary lung cancer (predominantly lung adenocarcinoma) or breast cancer as their primary malignancy; 22 patients (19.3%) have miscellaneous malignancies as their primary cancer. A higher percentage of solitary BM was present in the subcohort undergoing upfront craniotomy plus tumor resection.

**Table 1:** Patient demographics, tumor, disease, and treatment-related characteristics in the 114 patients with newly diagnosed brain oligometastases.

Characteristics	Entire cohort of brain oligometastases (*n* = 114)	Surgical cavities (*n* = 85)	Intact metastases (*n* = 29)	*p*-value
**Age at registration**				0.32
Mean (SD)	57.4 (9.7)	56.5 (8.9)	60.0 (11.3)	
Median (IQR)	57.3 (12.9)	56.6 (14.0)	59.7 (9.9)	
Range	26.7–83	28.30–77.20	26.70–83.00	
<65	90 (78.95%)	69 (81.18%)	21 (72.41%)	
≥65	24 (21.05%)	16 (18.82%)	8 (27.59%)	
**Gender**				0.59
Female	60 (52.6%)	46 (54.1%)	14 (48.3%)	
Male	54 (47.4%)	39 (45.9%)	15 (51.7%)	
**KPS performance status**				0.022*
KPS ≥ 90	50 (43.9%)	32 (37.65%)	18 (62.1%)	
70 ≤ KPS < 90	64 (56.1%)	53 (62.35%)	11 (37.9%)	
**ECOG performance status**				0.016*
0–1	93 (81.6%)	65 (76.5%)	28 (96.55%)	
2	21 (18.4%)	20 (23.5%)	1 (3.45%)	
**Baseline Barthel index**				0.37
Mean (SD)	92.59 (15.83)	91.24 (17.52)	96.55 (8.25)	
Median (IQR)	100 (5)	100 (10.0)	100 (2.5)	
Full, 100	79 (69.3%)	57 (67.1%)	22 (75.9%)	
<100	35 (30.7%)	28 (32.9%)	7 (24.1%)	
**Baseline FIM scale**				0.45
Mean (SD)	117.74 (16.00)	116.28 (17.54)	122.00 (9.25)	
Median (IQR)	125 (10)	125 (12)	126 (2)	
Full, 126	56 (49.1%)	40 (47.1%)	16 (55.2%)	
<126	58 (50.9%)	45 (52.9%)	13 (44.8%)	
**Histological subtype of primary cancer**				0.004*
Lung, adenocarcinoma	64 (56.1%)	41 (48.2%)	23 (79.3%)	
EGFR mutant	36 (31.6%)	23 (27.1%)	13 (44.8%)	
EGFR wild type	26 (22.8%)	16 (18.8%)	10 (34.5%)	
ALK mutation	2 (1.8%)	2 (2.4%)	0 (0.0%)	
Lung, non-adenocarcinoma	11 (9.7%)	7 (8.2%)	4 (13.8%)	
Breast	17 (14.9%)	17 (20.0%)	0 (0.0%)	
Her-2 overexpression	6 (5.3%)	6 (7.1%)	0 (0.0%)	
Triple negative	8 (7.0%)	8 (9.4%)	0 (0.0%)	
ER or PR positive	3 (2.6%)	3 (3.5%)	0 (0.0%)	
Others^a^	22 (19.3%)	20 (23.5%)	2 (6.9%)	
**Number of brain metastatic lesions**				0.07
Solitary	71 (62.3%)	57 (67.1%)	14 (48.3%)	
2–3	43 (37.7%)	28 (32.9%)	15 (51.7%)	
**The technique of SIB**				0.022*
Not applied	64 (56.1%)	53 (62.35%)	11 (37.9%)	
Attempted	50 (43.9%)	32 (37.65%)	18 (62.1%)	
**Status/control of extracranial disease**				0.018*
Stable or controlled	26 (22.8%)	24 (28.2%)	2 (6.9%)	
Uncontrolled yet	88 (77.2%)	61 (71.8%)	27 (93.1%)	
**RTOG RPA class**				0.012*
Class I	22 (19.3%)	21 (24.7%)	1 (3.45%)	
Class II	92 (80.7%)	64 (75.3%)	28 (96.55%)	

HS-WBRT, hippocampus-sparing whole-brain radiotherapy; KPS, Karnofsky performance status; ECOG, Eastern Cooperative Oncology Group; FIM, functional independence measure; EGFR, epidermal growth factor receptor; ALK, anaplastic lymphoma kinase; Her-2, human epidermal growth factor receptor; ER, estrogen receptor; PR, progesterone receptor; SIB, simultaneous integrated boost; RTOG RPA class, Radiation Therapy Oncology Group recursive partitioning analysis.

a The 22 patients with other histology type including hepatocellular carcinoma (4), colorectal cancer (3), esophageal cancer (2), unknown primary cancer (2), sarcoma (2), neuroendocrine carcinoma of uterine cervix (1), embryonal carcinoma (1), malignant melanoma (1), endometrial adenocarcinoma (1), urinary bladder cancer (1), renal cell carcinoma (3), and gastric cancer (1). *Asterisk symbols indicate statistically significant values (p < 0.05).

### OS and CNS Progression After HS-WBRT

There were eighty-eight deaths (77.2%) at the end of the study. Among the deceased patients, 85 patients (74.6%) died of cancer-related causes of death, and 31 (27.2%) died of neurologic causes directly resulting from their BM per se. However, 66 (57.9%) cancer-specific deaths were attributed to non-neurologic causes associated with systemic (extracranial) progression.

**Figure 1:**
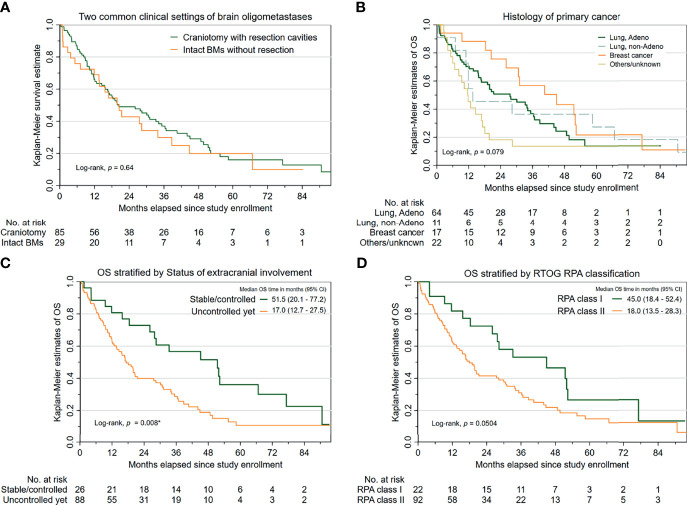
Survival outcomes in the 114 cancer patients with newly diagnosed brain oligometastases managed with HS-WBRT. **(A)** Kaplan–Meier estimates of overall survival (OS), stratified into two common clinical contexts (intact BMs versus resection cavities). **(B)** K-M estimates of OS, stratified by the histopathology of the primary malignancy. **(C)** K-M estimates of OS, stratified by the status of extracranial involvement. **(D)** K-M estimates of OS, based on the RTOG RPA classification.

Regarding Kaplan–Meier, estimates of OS are portrayed in **Figure 1A**. There was no significant difference in OS between two subcohorts with oligometastatic brain disease (patients with resection cavities post-craniotomy versus those with intact BMs). Additionally, **Table 2** reveals that several characteristics that may pose increased hazards to OS in patients undergoing HS-WBRT included suboptimal performance status (KPS < 90 or ECOG worse than 1) before receiving HS-WBRT, diminished activities of daily living (indicated by Barthel index or FIM scale), histological subtype of the primary cancer (**Figure 1B**), and the status of extracranial involvement at recruitment (**Figure 1C**). Similarly, it appears that the prognostic classification grouping according to RTOG Recursive Partitioning Analysis also conveys survival impact (**Figure 1D**). After controlling for these clinically significant characteristics, the impact of craniotomy plus tumor resection (versus intact BMs) on OS still failed to be statistically significant (adjusted HR, 1.257; 95% CI, 0.725–2.182; *p* = 0.415) (**Table 2**).

**Table 2:** Two patterns of cancer-specific deaths based on competing-risks analyses.

Covariate/parameter	OS		Neurologic death			Non-neurologic death	
	Crude HR (95% CI)	Adjusted HR (95% CI)	Crude HR (95% CI)		aHR (95% CI)	Crude HR (95% CI)	aHR (95% CI)
	*p*-value	*p*-value	*p*-value		*p*-value	*p*-value	*p*-value
**Subcohort**							
Surgical cavities	1	Reference	1	Reference		1	Reference
Intact metastases	1.117 (0.688–1.812)	1.257 (0.725–2.182)^a^	0.082 (0.011–0.595)	0.130 (0.019–0.898)^b^		1.778 (1.076–2.940)	1.883 (1.065–3.329)^b^
	*p* = 0.65	*p* = 0.42	*p* = 0.013	***p* = 0.039**		*p* = 0.025	***p* = 0.026**
PCI	0.626 (0.271–1.446)	Not analyzed	NA	NA		NA	NA
	*p* = 0.273						
**Age at enrollment**							
A continuous variable	0.984 (0.963–1.006)		0.981 (0.943–1.021)			0.990 (0.967–1.014)	
	*p* = 0.15		*p* = 0.35			*p* = 0.40	
**Gender**							
Female	1		1			1	
Male	0.816 (0.536–1.243)		1.601 (0.760–3.373)			0.790 (0.491–1.272)	
	*p* = 0.34		*p* = 0.22			*p* = 0.33	
**KPS just before the HS-WBRT** course							
KPS ≥ 90	1	Reference	1		Reference	1	Reference
70 ≤ KPS < 90	1.470 (0.956–2.260)	1.563 (0.943–2.260)	1.601 (0.760–3.373)		1.148 (0.536–3.373)	1.152 (0.712–1.866)	1.006 (0.534–1.866)
	*p* = 0.08	*p* = 0.08	*p* = 0.22		*p* = 0.72	*p* = 0.56	*p* = 0.99
**Baseline ECOG performance status**							
0–1	1	Reference	1		Reference	1	Reference
2	1.556 (1.065–2.273)	1.278 (0.674–2.273)	1.756 (0.877–3.516)		1.535 (0.613–3.516)	1.636 (1.030–2.601)	1.026 (0.372–2.601)
	*p* = 0.022	*p* = 0.45	*p* = 0.11		*p* = 0.36	*p* = 0.037	*p* = 0.96
**Barthel index**							
A continuous variable	0.983 (0.970–0.995)		1.002 (0.974–1.031)			0.978 (0.964–0.993)	
	*p* = 0.006		*p* = 0.90			*p* = 0.004	
Full, 100	1	Reference	1		Reference	1	Reference
<100	1.662 (1.071–2.580)	1.765 (1.121–2.580)	0.723 (0.323–1.618)		0.741 (0.325–1.692)	2.147 (1.302–3.542)	2.361 (1.424–3.542)
	*p* = 0.023	***p* = 0.014**	*p* = 0.43		*p* = 0.48	*p* = 0.003	***p* = 0.001**
**FIM scale**							
A continuous variable	0.983 (0.971–0.996)		0.999 (0.974–1.025)			0.978 (0.963–0.994)	
	*p* = 0.010		*p* = 0.964			*p* = 0.007	
Full, 126	1	Reference	1		Reference	1	Reference
<126	1.204 (0.790–1.838)	1.324 (0.859–1.838)	0.651 (0.323–1.312)		0.646 (0.294–1.312)	1.720 (1.062–2.785)	1.923 (1.159–2.785)
	*p* = 0.39	*p* = 0.20	*p* = 0.23		*p* = 0.28	*p* = 0.027	***p* = 0.011**
**Histological subtype of primary cancer**							
Lung, adenocarcinoma	1	Reference	1		Reference	1	Reference
Lung, non-adenocarcinoma	0.905 (0.443–1.848)	1.054 (0.507–1.848)	1.755 (0.483–6.374)		1.721 (0.440–6.374)	1.086 (0.517–2.280)	1.105 (0.509–2.280)
	*p* = 0.78	*p* = 0.89	*p* = 0.39		*p* = 0.44	*p* = 0.83	*p* = 0.80
Breast	0.677 (0.363–1.262)	0.986 (0.462–1.262)	4.445 (1.964–10.062)		2.118 (0.731–10.062)	0.566 (0.286–1.119)	1.135 (0.509–1.119)
	*p* = 0.22	*p* = 0.97	*p* < 0.0001		*p* = 0.17	*p* = 0.10	*p* = 0.76
Others or unknown	1.659 (0.966–2.850)	1.947 (1.004–2.850)	3.653 (1.385–9.640)		2.506 (0.869–9.640)	0.920 (0.443–1.913)	1.003 (0.373–1.913)
	*p* = 0.067	***p* = 0.049**	*p* = 0.009		*p* = 0.09	*p* = 0.50	*p* = 0.99
**Whether the primary cancer was lung adenocarcinoma or not**							
Lung adenocarcinoma	1	Reference	1		Reference	1	Reference
All others	1.018 (0.663 – 1.562)	1.276 (0.761 – 2.140)	3.432 (1.597–7.375)		2.647^c^ (1.209–5.795)	0.810 (0.495–1.325)	0.749^c^ (0.420–1.335)
	*p* = 0.94	*p* = 0.36	*p* = 0.002		***p* = 0.015**	*p* = 0.40	*p* = 0.33
**Number of brain metastatic lesions**							
Solitary	1	Reference	1		Reference	1	Reference
2–3 metastatic foci	1.165 (0.754–1.801)	1.246 (0.782–1.801)	0.816 (0.388–1.717)		1.084 (0.513–1.717)	1.178 (0.730–1.900)	1.080 (0.642–1.900)
	*p* = 0.49	*p* = 0.36	*p* = 0.59		*p* = 0.83	*p* = 0.50	*p* = 0.77
**Extent of resection**							
En-bloc gross total	1		1			1	
Less than gross total	1.221 (0.555–2.687)		1.506 (0.553–4.102)			1.352 (0.496–3.686)	
	*p* = 0.62		*p* = 0.42			*p* = 0.56	
**The technique of SIB**							
Not applied	1		1			1	
Attempted	1.154 (0.743–1.793)		0.491 (0.219–1.098)			1.256 (0.770–2.049)	
	*p* = 0.52		*p* = 0.08			*p* = 0.36	
**Status and control of extracranial disease**							
Stable or controlled	1	Reference	1		Reference	1	Reference
Untreated yet or uncontrolled	2.005 (1.180–3.406)	2.111 (1.128–3.406)	0.340 (0.174–0.663)		0.588^d^ (0.255–1.357)	4.137 (1.937–8.836)	3.788^d^ (1.615–8.885)
	*p* = 0.010	***p* = 0.019**	*p* = 0.002		*p* = 0.21	*p* < 0.0001	***p* = 0.002**
**RTOG RPA class**							
Class I	1		1			1	
Class II	1.734 (0.992–3.031)		0.344 (0.175–0.675)			4.677 (1.942–11.262)	
	*p* = 0.053		*p* = 0.002			*p* = 0.001	

OS, overall survival; PCI, primary cranial irradiation; aHR, adjusted hazard ratio; KPS, Karnofsky performance status; HS-WBRT, hippocampus-sparing whole-brain radiotherapy; ECOG, Eastern Cooperative Oncology Group; FIM, functional independence measure; SIB, simultaneous integrated boost; RTOG RPA, Radiation Therapy Oncology Group recursive partitioning analysis.

a Adjusted to control for the other five covariates which are also clinically significant or relevant: 1) age at enrollment, 2) baseline ECOG performance status just before the HS-WBRT course, 3) histological subtype, 4) number of brain metastatic lesions, and 5) status of extracranial disease.

b Adjusted by the other four covariates which are considered clinically relevant or significant: 1) age at enrollment, 2) histological subtype, 3) ECOG performance status, and 4) status of extracranial disease.

c Adjusted to control for the other three clinically important characteristics: 1) age at enrollment, 2) baseline ECOG performance status just before the HS-WBRT course, and 3) the two major clinical contexts of arranging the course of HS-WBRT (intact BMs versus resection cavities post-craniotomy).

d Adjusted by the other four covariates which are assumed clinically relevant or significant: 1) age at enrollment, 2) histological subtype, 3) ECOG performance status, and 4) the two major clinical contexts of administering the HS-WBRT course (intact BMs versus resection cavities post-craniotomy). NA, Not Applicable. Bold letters indicate statistically significant values (p < 0.05).

### Oncological Outcomes and Competing-Risks Analyses

**Figure 2:**
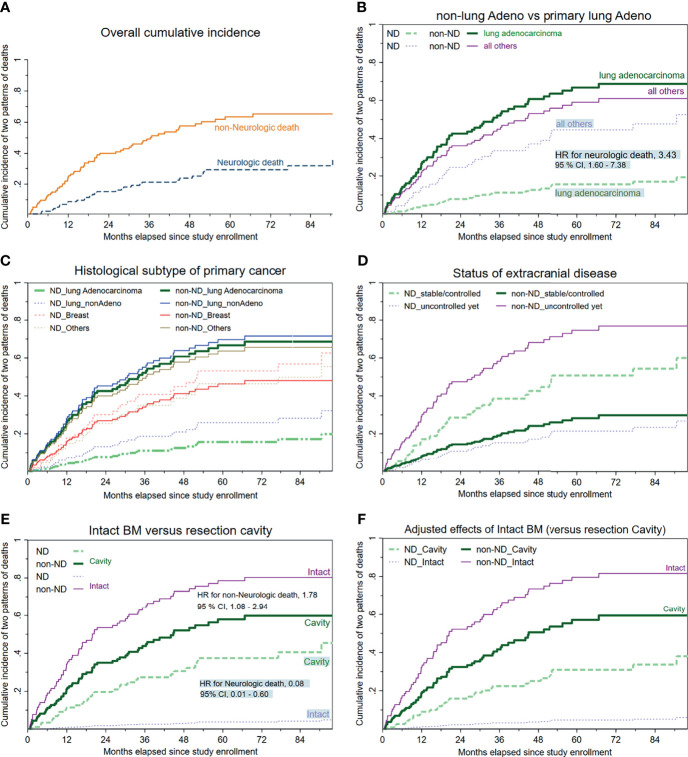
Cumulative incidence curves of neurologic/non-neurologic death, stratified by available clinical characteristics. **(A)** Overall cumulative incidence curves regarding neurologic and non-neurologic death. **(B)** Histology of primary malignancy categorized as a binary covariate (primary lung adenocarcinoma versus all others). **(C)** Histology of primary cancer stratified into four subgroups, with lung adenocarcinoma as the reference group. **(D)** Status/control of extracranial disease. **(E)** Cumulative incidence of death depending on whether upfront craniotomy plus tumor resection was performed. **(F)** The mutual associations between the clinical setting (intact BM versus resection cavity) and the two major cancer-specific causes of death (neurologic or non-neurologic) after controlling for the other meaningful covariates.

**Figure 2A** illustrates the overall cumulative incidence curves of neurologic death and non-neurologic death, respectively. The median time to neurologic death was 19.8 months (95% CI, 15.8 to 23.8), whereas the median time to non-neurologic death was 15.6 months (95% CI, 19.2 to 35.1). At 12 months, the cumulative incidence rates of the two principal causes of death (which were not always mutually exclusive) were 8.77% for neurologic death and 24.6% for non-neurologic death, respectively.

As displayed in **Table 2**, competing-risks analyses disclosed that despite not always reaching statistical significance, the following clinical factors tended to be associated with increased or decreased likelihoods of neurologic death: breast cancer or miscellaneous malignancies apart from primary lung cancers (**Figures 2B, C**), an uncontrolled/untreated extracranial status (**Figure 2D**), and belonging to the subcohort with intact BMs (versus that with resection cavities post-craniotomy) at study enrollment (**Figure 2E**).

For HS-WBRT-treated patients, the clinical characteristics predisposing our patients who received HS-WBRT to an increased risk of dying from progressive systemic disease (non-neurologic death) include suboptimal activities of daily living, an uncontrolled/untreated status of extracranial involvement (**Figure 2D**), and being in the subgroup of intact BMs (**Figure 2E**) because no craniotomy was recommended or attempted. The competing-risks models are exhibited in **Tables 2** and **3**.

**Table 3:** All oncological outcomes addressed in the current study, including the competing-risks analyses tailored to CNS-CE as the first event and two patterns of cancer-specific death.

Covariate/parameter	OS	CNS-CE as first event	LMD recurrence	Neurologic death	Non-neurologic death
	Crude HR (95% CI)	Crude HR (95% CI)	Crude HR (95% CI)	Crude HR (95% CI)	Crude HR (95% CI)
	*p*-value	*p*-value	*p*-value	*p*-value	*p*-value
**Subcohort**						
Surgical cavities	1	1	1	1	1
Intact metastases	1.117 (0.688–1.812)	0.131 (0.018–0.972)	**0**	0.082 (0.011–0.595)	1.778 (1.076–2.940)
	*p* = 0.65	***p* = 0.047**	***p* < 0.001**	***p* = 0.013**	***p* = 0.025**
PCI	0.626 (0.271–1.446)	Not analyzed	NA	NA	NA
	*p* = 0.27				
**Age at enrollment**					
<65	1	1	1	1	1
≥65	*p* = 0.70 (0.408–1.216)	*p* = 0.91 (0.298–2.766)	*p* = 0.42 (0.054–3.259)	*p* = 0.19 (0.511–2.757)	*p* = 0.55 (0.286–1.039)
	*p* = 0.21	*p* = 0.87	*p* = 0.41	*p* = 0.69	*p* = 0.07
**Gender**									
Female	1	1	1	1	1
Male	0.816 (0.536–1.243)	1.395 (0.572–3.400)	2.101 (0.541–8.155)	1.601 (0.760–3.373)	0.790 (0.491–1.272)
	*p* = 0.34	*p* = 0.46	*p* = 0.28	*p* = 0.22	*p* = 0.33
**KPS performance status just before the HS-WBRT course**					
KPS ≥ 90	1	1	1	1	1
70 ≤ KPS < 90	1.470 (0.956–2.260)	2.741 (1.007–7.459)	1.180 (0.334–4.173)	1.601 (0.760–3.373)	1.152 (0.712–1.866)
	*p* = 0.08	**0.048**	*p* = 0.80	*p* = 0.22	*p* = 0.56
**ECOG performance status**					
0–1	1	1	1	1	1
2	1.556 (1.065–2.273)	1.896 (0.734–4.897)	3.013 (0.860–10.563)	1.756 (0.877–3.516)	1.636 (1.030–2.601)
	***p* = 0.022**	*p* = 0.19	*p* = 0.09	*p* = 0.11	***p* = 0.037**
**Barthel index**					
A continuous variable	0.983 (0.970–0.995)	0.980 (0.959–1.001)	0.996 (0.942–1.053)	1.002 (0.974–1.031)	0.978 (0.964–0.993)
	***p* = 0.006**	*p* = 0.07	*p* = 0.90	*p* = 0.90	***p* = 0.004**
Full, 100	1	1	1	1	1
<100	1.662 (1.071–2.580)	1.175 (0.472–2.921)	0.549 (0.116–2.601)	0.723 (0.323–1.618)	2.147 (1.302–3.542)
	***p* = 0.023**	*p* = 0.73	*p* = 0.45	*p* = 0.43	***p* = 0.003**
**FIM scale**					
A continuous variable	0.983 (0.971–0.996)	0.984 (0.962–1.006)	0.997 (0.947–1.050)	0.999 (0.974–1.025)	0.978 (0.963–0.994)
	***p* = 0.010**	*p* = 0.15	*p* = 0.92	*p* = 0.96	***p* = 0.007**
Full, 126	1	1	1	1	1
<126	1.204 (0.790–1.838)	0.710 (0.300–1.682)	0.622 (0.177–2.186)	0.651 (0.323–1.312)	1.720 (1.062–2.785)
	*p* = 0.39	*p* = 0.44	*p* = 0.46	*p* = 0.23	***p* = 0.027**
**Histological subtype of primary cancer**					
Lung adenocarcinoma	1	1	1	1	1
Lung non-adenocarcinoma	0.905 (0.443–1.848)	0.734 (0.087–6.200)	**0**	1.755 (0.483–6.374)	1.086 (0.517–2.280)
	*p* = 0.78	*p* = 0.78	***p* < 0.0001**	*p* = 0.39	*p* = 0.83
Breast	0.677 (0.363–1.262)	1.880 (0.599–5.905)	1.949 (0.359–10.583)	4.445 (1.964–10.062)	0.566 (0.286–1.119)
	*p* = 0.220	*p* = 0.279	*p* = 0.439	***p* < 0.0001**	*p* = 0.102
Others or unknown	1.659 (0.966–2.850)	3.608 (1.339–9.722)	3.113 (0.781–12.406)	3.653 (1.385–9.640)	0.920 (0.443–1.913)
	***p* = 0.07**	***p* = 0.011**	*p* = 0.11	***p* = 0.009**	*p* = 0.50
**Number of brain metastatic lesions at diagnosis**					
Solitary	1	1	1	1	1
2–3 metastatic foci	1.165 (0.754–1.801)	0.831 (0.338–2.042)	0.420 (0.090–1.966)	0.816 (0.388–1.717)	1.178 (0.730–1.900)
	*p* = 0.49	*p* = 0.69	*p* = 0.27	*p* = 0.59	*p* = 0.50
**Extent of resection**					
En-bloc gross total	1	1	1	1	1
Less than gross total	1.221 (0.555–2.687)	1.735 (0.547–5.503)	0	1.506 (0.553–4.102)	1.352 (0.496–3.686)
	*p* = 0.62	*p* = 0.35		*p* = 0.42	*p* = 0.56
**The technique of SIB**					
Not applied	1	1	1	1	1
Attempted	1.154 (0.743–1.793)	0.494 (0.191–1.273)	0.549 (0.142–2.118)	0.491 (0.219–1.098)	1.256 (0.770–2.049)
	*p* = 0.52	*p* = 0.14	*p* = 0.38	*p* = 0.08	*p* = 0.36
**Status and control of extracranial disease**					
Stable or controlled	1	1	1	1	1
Uncontrolled yet	2.005 (1.180–3.406)	0.359 (0.153–0.841)	0.274 (0.080–0.931)	0.340 (0.174–0.663)	4.137 (1.937–8.836)
	***p* = 0.010**	***p* = 0.018**	***p* = 0.038**	***p* = 0.002**	***p* < 0.001**
**RTOG RPA class**					
Class I	1	1	1	1	1
Class II	1.734 (0.992–3.031)	0.362 (0.153–0.853)	0.343 (0.098–1.192)	0.344 (0.175–0.675)	4.677 (1.942–11.262)
	*p* = 0.053	***p* = 0.020**	*p* = 0.09	***p* = 0.002**	***p* = 0.001**

OS, overall survival; PCI, primary cranial irradiation; CNS-CE, central nervous system-composite endpoint; LMD, leptomeningeal disease; KPS, Karnofsky performance status; HS-WBRT, hippocampus-sparing whole-brain radiotherapy; ECOG, Eastern Cooperative Oncology Group; FIM, functional independence measure; SIB, simultaneous integrated boost; RTOG RPA, Radiation Therapy Oncology Group recursive partitioning analysis. NA, Not Applicable. Bold letters indicate statistically significant values (p < 0.05).

**Figures 2E, F** show the differences between the two distinct scenarios (intact BMs versus cavities post-craniotomy in probabilities of neurologic/non-neurologic deaths). Owing to the mutually exclusive nature of competing-risks effects exerted by either neurologic or non-neurologic death, the predominantly high incidence of non-neurologic death, which usually occurred earlier in the subcohort with intact BMs, apparently rendered those with intact BMs relatively unlikely to die of neurologic death. Specifically, the patients with intact BMs were significantly more likely to die from progressive extracranial disease (HR, 1.78; 95% CI, 1.08–2.95; *p* = 0.025) compared with those with resection cavities post-craniotomy. Consequently, patients with intact BMs were unlikely to survive long enough to encounter the threats of neurologic death (HR, 0.08, 95% CI, 0.01–0.60; *p* = 0.013, **Figure 2E**) than those with surgical cavities post-craniotomy.

After being adjusted to control for other clinically relevant characteristics, the adjusted HR for non-neurologic death in the subcohort of intact BMs was 1.88 times higher than in the subcohort with resection cavities post-craniotomy (adjusted HR, 1.88; *p* = 0.026; **Figure 2F**). In addition, adjusted HRs of clinical characteristics predicting neurologic/non-neurologic death were computed iteratively after controlling for other available covariates (**Table 2**).

**Figure 3:**
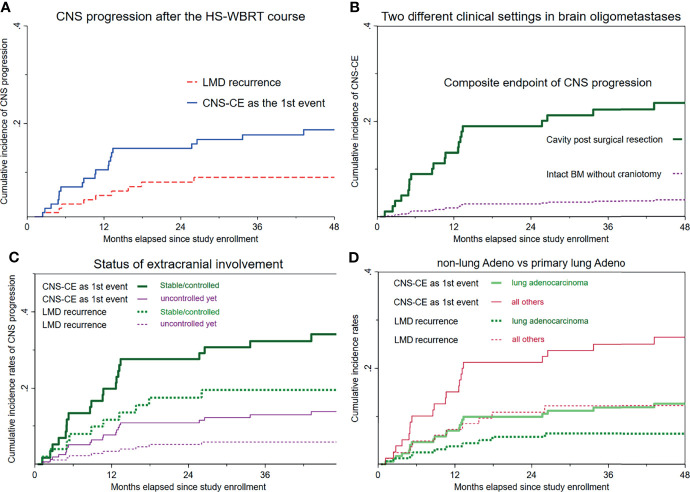
Cumulative incidence curves of LMD recurrence and the composite endpoint of CNS progression as the first event. **(A)** Overall cumulative incidence curves of LMD recurrence and CNS-CE (*n* = 114). **(B)** Cumulative incidence curves of CNS-CE as the first event, stratified according to whether upfront craniotomy was performed. **(C)** Both cumulative incidence curves, stratified based on the status of extracranial involvement. **(D)** Cumulative incidence rates according to primary lung adenocarcinoma versus all others.

LMD recurrence after HS-WBRT was observed clinically and radiographically in all of the 114 patients. **Figure 3A** illustrates that the cumulative incidence of CNS progression manifesting as LMD recurrence after HS-WBRT was 5.26% at 12 months, 7.98% at 18 months, and 7.98% at 24 months. The median time to develop LMD occurrence in the 10 patients was only 9.77 months from the date of being enrolled. Likewise, the cumulative incidence of the composite endpoint of CNS progression (CNS-CE) as the first event is also illustrated in the same plot (**Figure 3A**).

As mentioned above, dying from cancer-specific causes of death, which was assumed non-neurologic, would usually manifest considerably earlier than would any event resulting from CNS progression. Therefore, the CNS-CE as the first event was computed, accounting for the competing risks of either non-CNS progression or death, whichever happened first. Overall, the median time to such event was only 10.6 months from the date of being enrolled. The cumulative incidence rate of CNS-CE as the first event in the entire cohort of 114 patients was 10.5% at 12 months, 14.9% at 24 months, 17.7% at 3 years, and 18.7% at 4 years (**Figure 3A**).

**Figure 3B** and **Supplementary Figure 1** illustrate the remarkable differences in either CNS-CE as the first event or LMD recurrence after undergoing HS-WBRT. CNS-CE as the first event was 23.8% in the subcohort with surgical cavities and only 3.49% in the subcohort with intact BMs (hazard ratio, 0.13; *p* = 0.047; **Figure 3B**), and no LMD recurrence was found in patients with intact BMs (**Table 3**). Moreover, as illustrated in **Figure 3C** **,** under the substantial impacts exerted by non-neurologic death, which usually manifests early and fatally, the associations between the initial status of extracranial involvement and neuro-oncological outcomes (either LMD recurrence or CNS-CE as the first event) were similar. Lastly, as for the association between histopathology of primary malignancy and the oncological outcomes of concern, it seemed that there was a tendency toward increased likelihoods of developing CNS progression if the primary malignancy was not of lung adenocarcinoma origin (HR for CNS-CE as first event, 2.28; 95% CI, 0.95–5.45; *p* = 0.065; **Figure 3D**). Although not statistically significant, some clinical characteristics appeared to be associated with the occurrence of CNS-CE without pre-existing non-CNS progression or death (**Table 3**, **Supplementary Figures 1** **,** **2**, and **Figure 3**).

## Discussion

In this prospective neurocognitive study, satisfactory oncological outcomes were observed through hippocampus sparing during the WBRT in the treatment of cancer patients who had brain oligometastases and a fair to good performance status. The important oncological outcomes, such as cumulative incidence of LMD recurrence and neurologic (or non-neurologic) cancer-specific death while treated with HS-WBRT, can greatly help neuro-oncologists in managing patients with oligometastatic brain disease.

The fundamental issue underlying cancer-related death is predicting which patients who have undergone HS-WBRT die from their brain oligometastases. Focusing on oncological outcomes-directed analyses would assist us in clarifying whether some certain cancer patients with brain oligometastases have an overwhelmingly increased risk of non-neurologic death despite the benefit in intracranial control achieved through WBRT. Additionally, the impact exerted by neurologic death may be substantially outweighed by death related to the overwhelming competing-risk event of non-neurologic death (9, 10). Non-neurologic death per se is more likely to be a challenging threat associated with progressive systemic disease to neuro-oncologists when treating cancer patients with brain oligometastases.

Consistent with the US large-scale retrospective studies (9, 10), our findings reinforced their reports that lung cancer patients were more likely to die from non-neurologic causes. It was thus concluded that a particularly higher risk of non-neurologic death occurring in patients with lung cancer might reflect the fact that further advance in effective systemic therapies was persistently deserved.

HS-WBRT is a local treatment whose effect is assumed to be local rather than systemic, and its target volume is tailored to the entire intracranial space. An outcome measure like OS may not adequately reflect the treatment effect of HS-WBRT. Therefore, neuro-oncological outcomes involving the concept of CNS progression have been emphasized (14).

Actually the occurrence of leptomeningeal spreading is not common, but CNS progression manifesting as LMD recurrence is usually devastating, leading to neurologic death inevitably (15). Regarding CNS progression manifesting as LMD after adjuvant brain irradiation after craniotomy, there are limited data reported directly on the subgroup receiving post-craniotomy adjuvant WBRT (16). By contrast, concerning LMD recurrence after postoperative SRS irradiating the resection cavity focally, its cumulative incidence is an increasingly recognized issue (17). Surgical resection of BMs per se is a risk factor predisposing cancer patients harboring cavities post-craniotomy to the manifestation of LMD recurrence, compared with those receiving upfront SRS/SRT tailored to the metastatic lesion(s) without immediate WBRT (16, 18). Interestingly, the only independent predictor for LMD recurrence was surgical resection per se after controlling for other confounders (18).

Similarly, we found that all events associated with LMD recurrence were present exclusively in the subcohort with resection cavities post-craniotomy, but not in the subcohort with intact BMs. Two rational explanations may account for such a drastic difference in the cumulative incidence of LMD recurrence between subcohorts. First, the consistent delivery of WBRT would naturally reduce the probability of leptomeningeal spreading, and this clinical benefit resulting from WBRT was documented in other studies (19, 20). Second, with intraoperative tumor spill during neurosurgical resection probably, the margins around the leptomeninges could adversely be the origins of CNS progression that cannot be eradicated completely by WBRT (21).

The majority of our results and discussion focused on oncological outcomes including CNS progression and cancer-related causes of death (non-neurologic, neurologic, respectively) based considerably on a competing-risks approach. It was acknowledged that neuro-oncological outcomes were indeed the main research aim addressed in the current manuscript. All of our patients received their course of whole-brain irradiation relying on the technique of hippocampus-sparing whole-brain radiotherapy which was designed and delivered in a standardized and consistent fashion, and we believe our study purpose and niche are innovative, which can easily be distinguished from past histological research. First, the so-called whole-brain irradiation used in most of the histological studies was traditional and conventional WBRT, which were basically bilateral opposed fields without utilizing the modern techniques of intensity-modulated radiotherapy or volume-modulated arc therapy. Second, the oncological outcomes addressed in the previous histological studies were mainly classical survival analyses such as Kaplan–Meier survival probability estimation and Cox proportional hazards regression model analyses. It was acknowledged that additional comprehensive analyses relying on a competing-risks approach were utilized in our study, attempting to explore and clarify the cancer-specific causes of death (neurologic or non-neurologic, mutually exclusive) and ultimately the more specific neuro-oncological endpoints including CNS progression measured as a composite endpoint (CNS-CE) and CNS progression manifesting as LMD recurrence.

The study had no stereotyped control group managed with conventional WBRT without the strategy of hippocampus sparing; however, we consider it unethical to conduct a prospective study with a control group allocated to conventional WBRT without hippocampus sparing. We consider that HS-WBRT indeed contributed to comparable neuro-oncological outcomes and superior neurocognitive outcomes. Moreover, two relevant randomized controlled trials (RCTs) integrated with a control arm of WBRT without hippocampal avoidance was reported in 2020 (22, 23). We also acknowledge that the confounding effect of molecular target therapy or immunotherapy was not included in our research protocol. Frankly speaking, this study was launched initially by neurosurgeons and conducted chiefly by radiation oncologists. The majority of our enrolled patients harboring newly diagnosed brain oligometastases at enrollment had not received molecular target therapy since they had no idea about their cancer diagnosis while being brought to an emergency department due to their active neurological symptoms.

Further prospective research comparing treatment effectiveness between HS-WBRT and upfront SRS/SRT without immediate WBRT is warranted. For patients with brain oligometastases, some neurosurgeons/physicians prefer upfront SRS/SRT, deferring WBRT until intracranial failure. By contrast, HS-WBRT would provide patients harboring brain oligometastases better intracranial tumor control without compromising neuropsychological outcomes than would upfront SRS without immediate WBRT.

Moreover, in this era of targeted therapy or immunotherapy, choosing among anticancer agents has been increasingly tailored to the molecular characterization in each distinct cancer individually. Previously, the challenges of unsatisfactory control over brain metastases have been attributed to a lack of CNS penetration of anticancer medication. Promisingly, recent studies have supported the role of certain targeted therapies in managing cancer patients with CNS metastases/progression based on the histopathology of primary malignancies (24, 25). In the future, it merits innovative research projects addressing and determining whether administration of HS-WBRT can be postponed until CNS progression has occurred or replaced by upfront SRS/SRT integrated into first-line systemic treatment with targeted therapy for managing brain oligometastases without leptomeningeal spreading.

## Conclusions

HS-WBRT has achieved favorable oncological outcomes by restricting the dose irradiating the hippocampus during WBRT. Oncological outcomes including neurologic/non-neurologic death after the HS-WBRT differ greatly between patients with intact BMs and those with cavities post-craniotomy, indicating they are clinically distinct subgroups; patients with resection cavities had a significantly increased likelihood of CNS progression (local failure, distant brain recurrence, or LMD recurrence) which might be associated with neurologic death eventually.

## Data Availability Statement

The original contributions presented in the study are included in the article/**Supplementary Material**. Further inquiries can be directed to the corresponding authors.

## Ethics Statement

The studies involving human participants were reviewed and approved by Chang Gung Medical Foundation Institutional Review Board, Taoyuan, Taiwan, and the IRB numbers are IRB 101-4151B and 103-1090C. The patients/participants provided their written informed consent to participate in this study.

## Author Contributions

S-YL, C-CC, and D-LT contributed to the study conception and design. S-YL, P-CP, C-LW, Y-MW, C-CL, C-HL, K-CW, and W-CC performed chart reviews for clinical data, follow-up, and data collection and maintained the clinical database. S-YL, C-CY, and D-LT did the data analyses, performed the statistical testing, and had access to the raw data. C-CY, C-CC, and K-CW provided the infrastructural support for this study. The manuscript was drafted by S-YL, edited by D-LT and C-CC, and submitted for comments to all contributing authors. All authors approved the final version of the manuscript.

## Funding

This study was supported by research grants (CMRPG3J0101, CMRPG2G0473, CMRPG3J1012) from Chang Gung Memorial Hospital at Linkou, Taoyuan, Taiwan, R.O.C.

## Conflict of Interest

The authors declare that the research was conducted in the absence of any commercial or financial relationships that could be construed as a potential conflict of interest.

## Publisher’s Note

All claims expressed in this article are solely those of the authors and do not necessarily represent those of their affiliated organizations, or those of the publisher, the editors and the reviewers. Any product that may be evaluated in this article, or claim that may be made by its manufacturer, is not guaranteed or endorsed by the publisher.
