# Supplementary material for: Oncological Outcomes After Hippocampus-Sparing Whole-Brain Radiotherapy in Cancer Patients With Newly Diagnosed Brain Oligometastases: A Single-Arm Prospective Observational Cohort Study in Taiwan
Source: Front Oncol. 2022 Jan 12;11:784635. doi: 10.3389/fonc.2021.784635 (PMC8790705; doi:10.3389/fonc.2021.784635)
Supplement: Supplementary file 1 [file DataSheet_1.docx]

Contents

[**Supplementary Figure 1**. Cumulative incidence rates of neuro-oncological outcomes after the HS-WBRT course. 2](#_Toc89350543)

[**Supplementary Figure 2.** Cumulative incidence curves of LMD recurrence and the composite endpoint of CNS progression (CNS-CE) as the first event, stratified by whether the primary malignancy was breast cancer or not. 3](#_Toc89350544)

**Supplementary Figure 1**. Cumulative incidence rates of neuro-oncological outcomes after the HS-WBRT course.

**
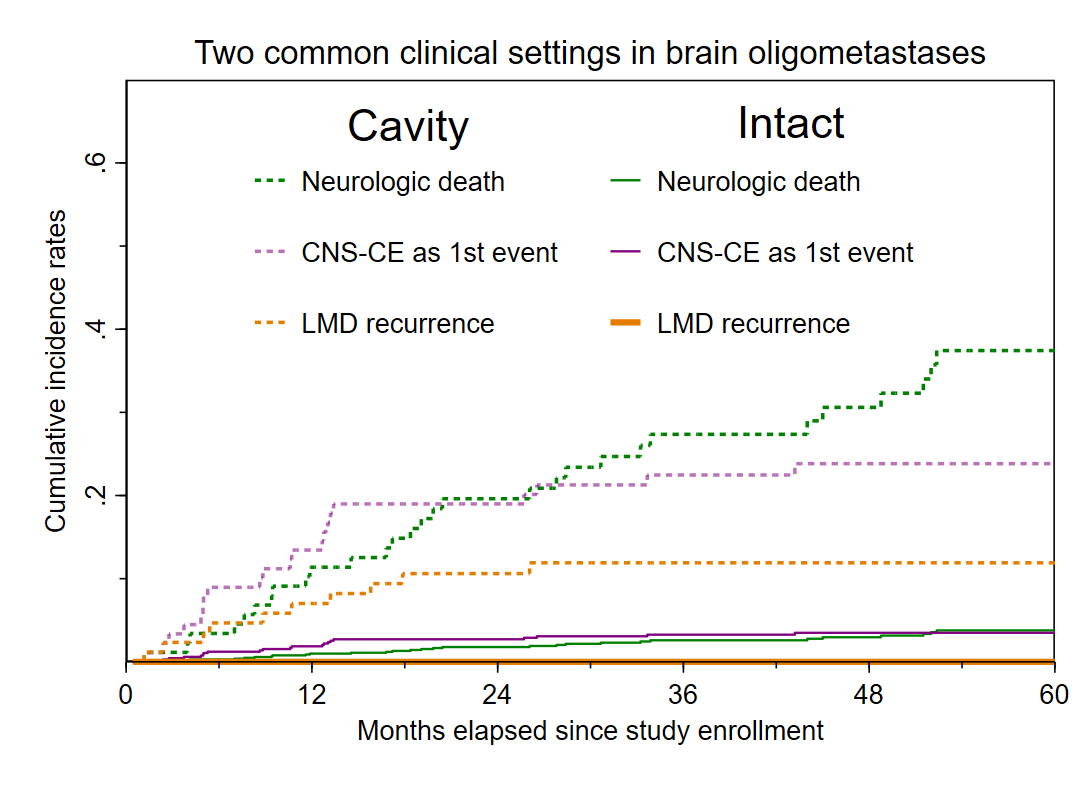
Abbreviations**

CNS-CE, Central Nervous System-Composite Endpoint; LMD, leptomeningeal disease.

**Supplementary Figure 2.** Cumulative incidence curves of LMD recurrence and the composite endpoint of CNS progression (CNS-CE) as the first event, stratified by whether the primary malignancy was breast cancer or not.

**
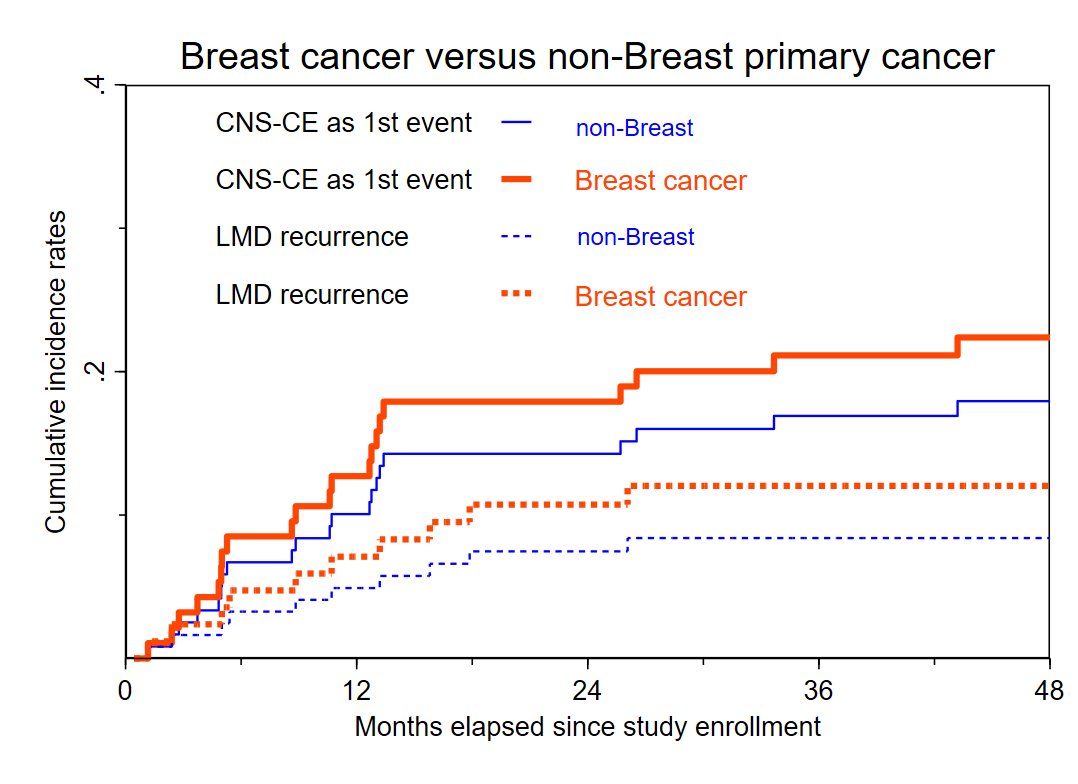
**
